# Bioinformatic analyses reveal the prognostic significance and potential role of ankyrin 3 (*ANK3*) in kidney renal clear cell carcinoma

**DOI:** 10.5808/gi.23013

**Published:** 2023-06-30

**Authors:** Keerakarn Somsuan, Siripat Aluksanasuwan

**Affiliations:** 1 School of Medicine, Mae Fah Luang University, Chiang Rai 57100, Thailand; 2 Cancer and Immunology Research Unit (CIRU), Mae Fah Luang University, Chiang Rai 57100, Thailand

**Keywords:** ankyrin 3 (*ANK3*), biomarker, kidney renal cell carcinoma, peroxisome proliferator-activated receptor, prognosis

## Abstract

Kidney renal clear cell carcinoma (KIRC) is one of the most aggressive cancer type of the urinary system. Metastatic KIRC patients have poor prognosis and limited therapeutic options. Ankyrin 3 (*ANK3*) is a scaffold protein that plays important roles in maintaining physiological function of the kidney and its alteration is implicated in many cancers. In this study, we investigated differential expression of *ANK3* in KIRC using GEPIA2, UALCAN, and HPA databases. Survival analysis was performed by GEPIA2, Kaplan-Meier plotter, and OSkirc databases. Genetic alterations of *ANK3* in KIRC were assessed using cBioPortal database. Interaction network and functional enrichment analyses of *ANK3*-correlated genes in KIRC were performed using GeneMANIA and Shiny GO, respectively. Finally, the TIMER2.0 database was used to assess correlation between *ANK3* expression and immune infiltration in KIRC. We found that *ANK3* expression was significantly decreased in KIRC compared to normal tissues. The KIRC patients with low *ANK3* expression had poorer survival outcomes than those with high *ANK3* expression. *ANK3* mutations were found in 2.4% of KIRC patients and were frequently co-mutated with several genes with a prognostic significance. *ANK3*-correlated genes were significantly enriched in various biological processes, mainly involved in peroxisome proliferator-activated receptor (PPAR) signaling pathway, in which positive correlations of *ANK3* with *PPARA* and *PPARG* expressions were confirmed. Expression of *ANK3* in KIRC was significantly correlated with infiltration level of B cell, CD8+ T cell, macrophage, and neutrophil. These findings suggested that *ANK3* could serve as a prognostic biomarker and promising therapeutic target for KIRC.

## Introduction

Kidney renal clear cell carcinoma (KIRC) or clear cell renal cell carcinoma is the most common histological type of kidney cancer, accounting for 70% of all cases [1]. According to global cancer statistics, there were 431,288 new cases and 179,368 new deaths for kidney cancer worldwide in 2020 [2]. Although localized KIRC can be cured by surgical treatment, patients frequently present with metastasis at diagnosis or develop recurrence after treatment, resulting in high mortality rate and limited therapeutic options [3,4]. Therefore, the identification of biomarkers is beneficial to improve diagnosis and prognosis for KIRC patients.

Ankyrin 3 (*ANK3*), also known as ankyrin G, belongs to ankyrin protein family. It is a scaffold protein that regulates the organization of membrane and cytoskeletal components [5]. *ANK3* is the most abundant ankyrin in kidney [6] and plays a crucial role in membrane assembly, epithelial cell polarization, and regulation of ion channels [7-9]. In cancers, genetic and expression alterations of *ANK3* have been reported in several studies [10-16]. Decreased *ANK3* expression was associated with poor survival outcome in prostate cancer [14] and androgen receptor‑positive breast cancer [15]. It has been shown that *ANK3* regulates cell cycle and inhibits cell invasion in prostate cancer cells [14]. Overexpression of *ANK3* promotes cell apoptosis and suppresses epithelial-mesenchymal transition in papillary thyroid carcinoma cells [16]. These findings indicate the prognostic value and tumor suppressive function of *ANK3* in cancers. Nevertheless, its prognostic significance and role in KIRC remain largely unknown.

In the present study, we performed an integrative bioinformatic analysis of molecular and clinical data from the publicly available datasets through various online databases. The expression of *ANK3* and its relationship to clinicopathologic outcomes in KIRC were explored in Gene Expression Profiling Interactive Analysis 2 (GEPIA2), University of ALabama at Birmingham CANcer data analysis portal (UALCAN), and Human Protein Atlas (HPA) databases. Prognostic significance of *ANK3* for KIRC was assessed by GEPIA2, Kaplan-Meier (KM) plotter, Online consensus Survival analysis for KIRC (OSkirc), and Tumor Immune Estimation Resource (TIMER) databases. *ANK3* mutations and co-mutations in KIRC were analyzed by the cBioPortal database. Potential roles of *ANK3* in KIRC carcinogenesis and immune infiltration were also investigated using GeneMANIA, Shiny GO, and TIMER2.0 databases.

## Methods

### Differential expression analysis

Differential expression of *ANK3* in KIRC compared to normal tissues was explored using GEPIA2 database (http://gepia2.cancer-pku.cn/) [17] and the UALCAN database (http://ualcan.path.uab.edu) [18]. The mRNA expression of *ANK3* was analyzed in KIRC (n = 533) and normal (n = 72) tissue samples in The Cancer Genome Atlas (TCGA) dataset using GEPIA2. The differential expression of ANK3 protein in KIRC (n = 110) and normal (n = 84) tissue samples was examined in the Clinical Proteomic Tumor Analysis Consortium (CPTAC) dataset using UALCAN. In addition, the protein expression levels of ANK3 in renal cancer and normal kidney tissues were explored in the HPA database (http://www.proteinatlas.org) [19,20].

### Analysis of the association of *ANK3* expression and clinicopathological features of KIRC patients

Relationships between *ANK3* mRNA expression and clinicopathological features, including age, race, sex, cancer stage, tumor grade, and nodal metastasis status in KIRC patients were analyzed in the TCGA dataset using the UALCAN database.

### Survival analysis

Survival analysis of *ANK3* expression in KIRC patients was performed using various databases, including GEPIA2, KM plotter (https://kmplot.com/analysis/) [21], OSKirc (https://bioinfo.henu.edu.cn/KIRC/KIRCList.jsp) [22]. In GEPIA2, patients (n=516) were split into low- and high-expression groups based on median expression value. Survival analysis by KM plotter was conducted for 530 KIRC patients. Low- and high-*ANK3* expression groups were divided using “Auto select best cut-off” option. For OSkirc, a total of 629 KIRC patients from combined data sources (TCGA, GSE22541, GSE29609, and GSE3) were subjected to survival analysis with the patients split by “upper 50%” option. The KM curves of overall survival of KIRC patients were plotted along with the log-rank p-value and hazard ratio (HR). Multivariable Cox proportional hazard regression analysis to assess an independent predictive value of *ANK3* expression was performed using the TIMER database (https://cistrome.shinyapps.io/timer/) [23,24].

### Genetic alteration analysis

Genetic alterations of *ANK3* were explored using cBioPortal for Cancer Genomics database (https://www.cbioportal.org/) [25,26]. The *ANK3* mutations and co-mutations in KIRC were analyzed in 1,496 samples in TCGA datasets (TCGA, Firehose Legacy; TCGA, Nature 2013; TCGA, PanCancer Atlas). Heatmap representing HR and the KM curve of *ANK3* co-mutated gene expression for overall survival of KIRC patients were created by GEPIA2.

### Interaction network and functional enrichment analyses of *ANK3*-correlated genes

The top 50 genes that were positively correlated with *ANK3* in KIRC based on Pearson correlation coefficient, were retrieved from GEPIA2 and subjected to further analyses. Interaction networks of *ANK3*-correlated genes were constructed using GeneMANIA (https://genemania.org/) [27]. Gene Ontology (GO) and Kyoto Encyclopedia of Genes and Genomes (KEGG) pathway enrichment analyses were performed and graphically visualized using ShinyGO (version 0.76.3) (http://bioinformatics.sdstate.edu/go/) [28]. The significance threshold for the enrichment was set at the false discovery rate ≤ 0.05.

### Analysis of relationship between *ANK3* and peroxisome proliferator-activated receptors in KIRC

Correlations of *ANK3* expression and peroxisome proliferator-activated receptors (PPARs), including PPARα (*PPARA*), PPARβ/δ (*PPARD*), and PPARγ (*PPARG*) expression in KIRC were analyzed in 516 samples using GEPIA2. KM curves of *PPARA*, *PPARD*, and PPRRG expressions for overall survival of KIRC patients were generated by GEPIA2. The protein expression levels of PPARA and PPARG in renal cancer and normal kidney tissues were explored in the HPA database.

### Immune infiltration analysis

Correlation between *ANK3* expression and abundance of tumor-infiltrating immune cells, including B cells, CD8+ T cells, CD4+ T cells, neutrophils, macrophages, and dendritic cells in KIRC was estimated by TIMER, TIDE, CIBERSORT, CIBERSORT-ABS, QUANTISEQ, XCELL, MCPCOUNTER, and EPIC algorithms through TIMER 2.0 database (http://timer.cistrome.org/) [29] with tumor purity adjustment. A heatmap representing the partial Spearman's correlation coefficient was plotted using GraphPad Prism version 8.0.1 (GraphPad Software, San Diego, CA, USA). Scatter plots of *ANK3* expression level and infiltration level of immune cells were visualized by the TIMER2.0 database.

### Statistical analysis

Differential expression analysis of *ANK3* was performed using one-way ANOVA in the GEPIA database and Student's t-test in the UALCAN database. Survival analysis was performed with the Kaplan-Meier method and log-rank test. Multivariate analysis was conducted by Cox’s proportional hazard model. Genetic alterations were analyzed by one-sided Fisher’s exact test in the cBioportal database. Pearson’s correlation analysis was used to evaluate the correlation between two genes expression. Correlation between *ANK3* expression and immune infiltration level was evaluated by the purity-adjusted partial Spearman’s correlation test. The p-value less than 0.05 was considered statistically significant.

## Results

### Differential expression of *ANK3* in KIRC and normal tissues

**Fig. 1.**
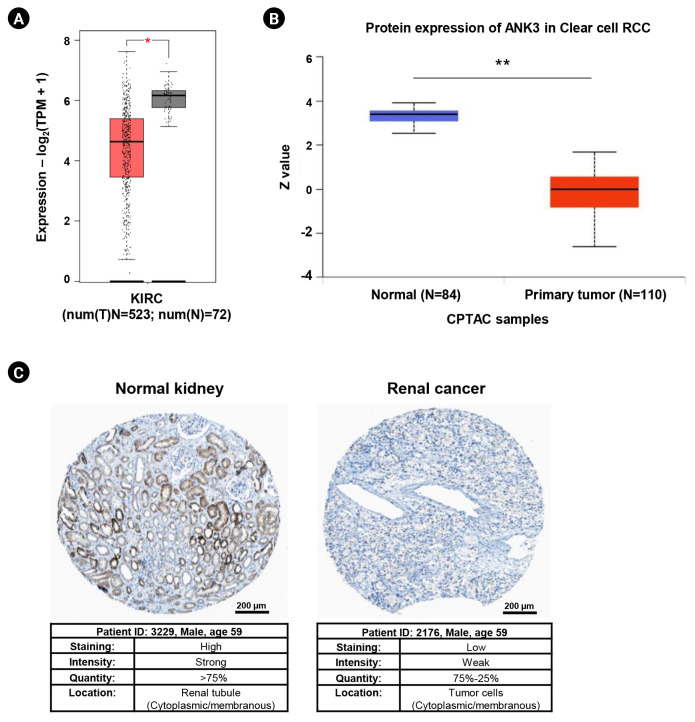
Expression of *ANK3* mRNA and protein in KIRC tissues. (A) Boxplot of *ANK3* mRNA expression in KIRC (red) and normal (grey) tissues from The Cancer Genome Atlas dataset (GEPIA2 database). (B) Boxplot of ANK3 protein expression in KIRC (red) and normal (blue) tissues from the CPTAC dataset (UALCAN database). (C) Representative immunohistochemical image of ANK3 protein expression in normal kidney and renal cancer tissues (HPA database). *ANK3*, ankyrin 3; KIRC, kidney renal clear cell carcinoma; GEPIA2, Gene Expression Profiling Interactive Analysis 2; CPTAC, Clinical Proteomic Tumor Analysis Consortium; UALCAN, University of ALabama at Birmingham CANcer data analysis portal; HPA, Human Protein Atlas; RCC, renal cell carcinoma; TPM, transcripts per million. ^*^p < 0.05, ^**^p < 0.01.

Differential expressions of *ANK3* in KIRC compared to normal tissue at mRNA and protein levels were investigated using GEPIA2 and UALCAN, respectively. GEPIA2 analysis showed that *ANK3* mRNA expression was significantly down-regulated in KIRC compared to normal tissues in the TCGA dataset (Fig. 1A). Similarly, a significant decrease of ANK3 protein expression in KIRC was observed from CPTAC dataset in UALCAN (Fig. 1B). Moreover, result from HPA database also demonstrated a decrease of ANK3 protein level in renal cancer compared to normal kidney tissues (Fig. 1C). These findings indicated that *ANK3* expression was significantly decreased in KIRC compared to normal tissues at both mRNA and protein levels.

### Association between *ANK3* expression and clinicopathological features of KIRC patients

**Fig. 2.**
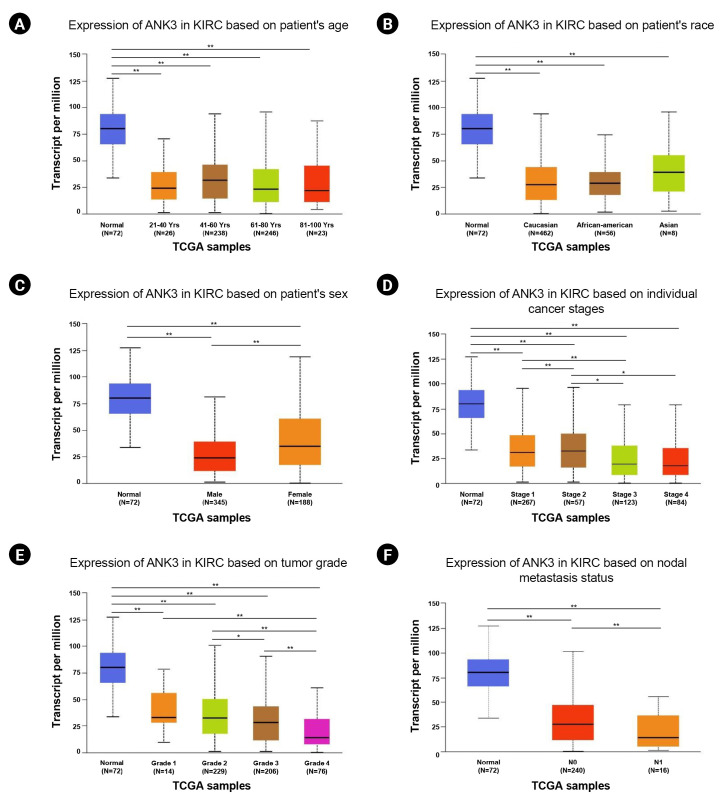
Relationship between *ANK3* mRNA expression and clinicopathological features in KIRC. Boxplot of *ANK3* mRNA expression in KIRC based on patient’s age (A), patient’s race (B), patient’s sex (C), individual cancer stage (D), tumor grade (E), and nodal metastasis status (F) from The Cancer Genome Atlas dataset (UALCAN database). *ANK3*, ankyrin 3; KIRC, kidney renal clear cell carcinoma; UALCAN, University of ALabama at Birmingham CANcer data analysis portal; TCGA, The Cancer Genome Atlas. ^*^p < 0.05, ^**^p < 0.01.

We assessed the associations between *ANK3* mRNA expression and clinicopathological features of KIRC patients using UALCAN. Based on the TCGA dataset, *ANK3* expression was not significantly associated with patient’s age and race (Fig. 2A and 2B). Male patients had a significantly lower level of *ANK3* expression compared to female patients (Fig. 2C). In addition, the data showed that *ANK3* expression was significantly correlated with cancer stage (Fig. 2D), tumor grade (Fig. 2E), and nodal metastasis status (Fig. 2F). These findings suggested that the decreased expression of *ANK3* may be a predictive indicator for KIRC severity and progression.

### Prognostic impact of *ANK3* expression in KIRC

**Fig. 3.**
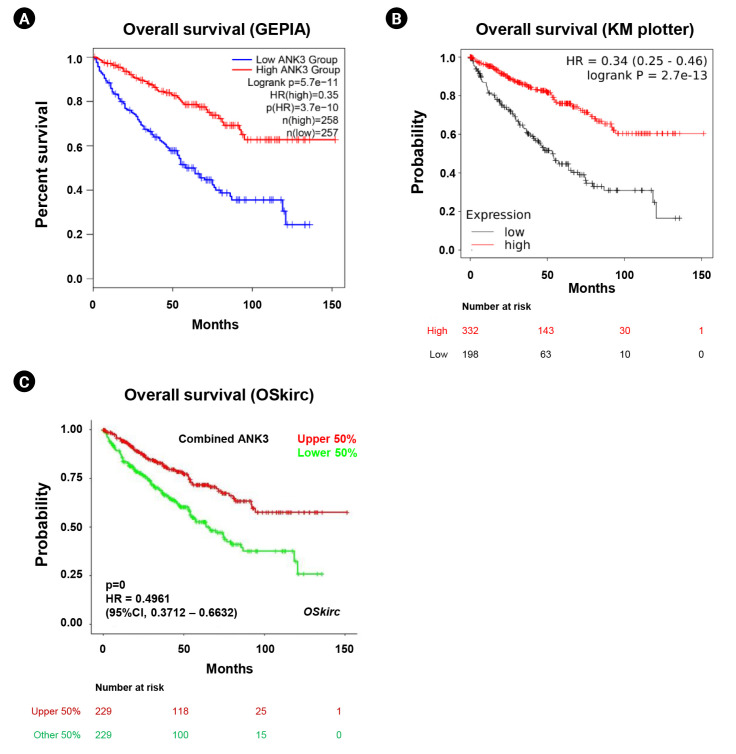
Relationship between *ANK3* mRNA expression and survival outcomes of KIRC patients. KM curves for overall survival in KIRC patients with low- and high-*ANK3* expression obtained from GEPIA2 (A), KM plotter (B), and OSkirc (C) databases. *ANK3*, ankyrin 3; KIRC, kidney renal clear cell carcinoma; KM, Kaplan-Meier; GEPIA2, Gene Expression Profiling Interactive Analysis 2; OSkirc, Online consensus Survival analysis for KIRC; HR, hazard ratio; CI, confidence interval.

We analyzed an association between ANK3 expression and overall survival of KIRC patients with low- and high-*ANK3* expression using GEPIA2, KM plotter, and OSkirc databases. The data from GEPIA2 showed that KIRC patients with low *ANK3* expression had significantly shorter overall survival than those with high *ANK3* expression (Fig. 3A). Significant associations of *ANK3* expression with overall survival of KIRC patients were consistently observed in KM plotter (Fig. 3B) and OSkirc (Fig. 3C) databases. In order to assess an independent predictive value of *ANK3* expression, multivariate analysis was performed using the TIMER database. The analysis results confirmed that *ANK3* expression was an independent prognostic factor for KIRC (Table 1). Thus, the low *ANK3* expression could indicate poor prognosis in KIRC patients.

### Genetic alteration of *ANK3* in KIRC

**Fig. 4.**
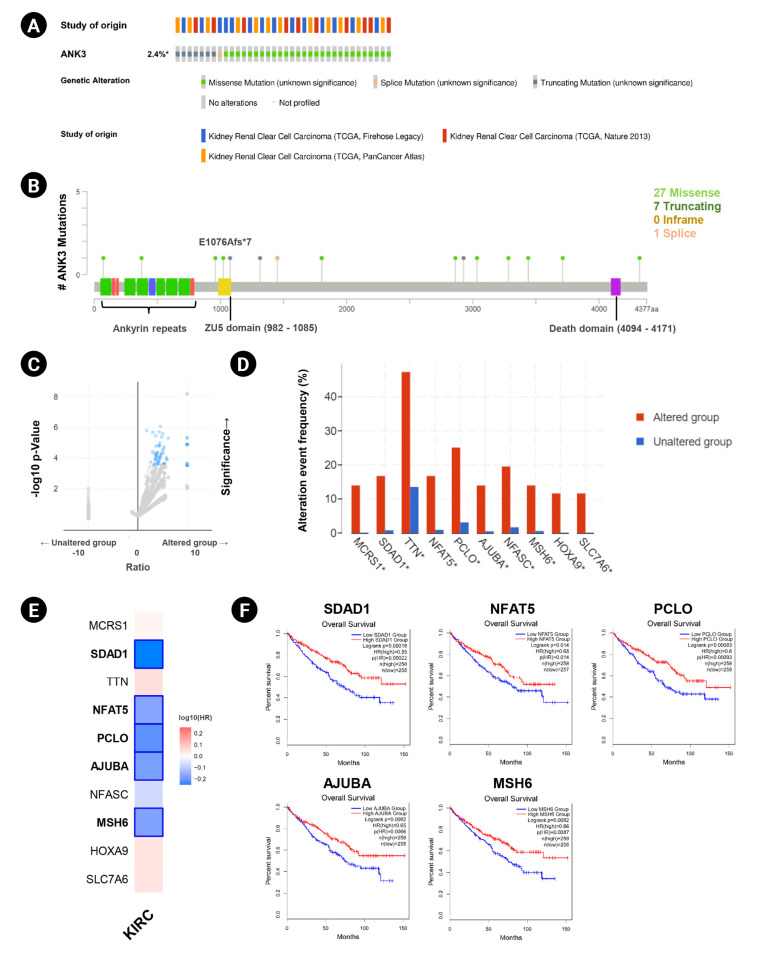
*ANK3* mutations and co-mutations in KIRC. (A) Genetic alteration frequency of *ANK3* in KIRC patients (cBioportal database). (B) Distribution of mutations along the *ANK3* gene in KIRC (cBioportal database). (C) Volcano plot of mutated genes in KIRC patients with and without *ANK3* alterations (cBioportal database). (D) Bar graph representing the alteration frequency of the top 10 genes with the most significantly co-mutated with *ANK3* in KIRC patients (cBioportal database). (E) Heatmap representing HR of each *ANK3* co-mutated gene for overall survival of KIRC patients (GEPIA2 database). (F) KM curves of *ANK3* co-mutated genes with a significant prognostic impact on overall survival for KIRC (GEPIA2 database). *ANK3*, ankyrin 3; KIRC, kidney renal clear cell carcinoma; GEPIA2, Gene Expression Profiling Interactive Analysis 2; KM, Kaplan-Meier; HR, hazard ratio.

Genetic alteration of *ANK3* in KIRC patients was analyzed using cBioPortal. Based on TCGA datasets, *ANK3* mutations were found in about 2.4% (36 of 1495 cases) of KIRC patients (Fig. 4A). There were 36 mutations distributed across the gene, in which missense mutations were the most frequent (28 of 36), followed by truncating (7 of 36) and splicing (1 of 36) mutations (Fig. 4B). In order to gain more insights into the underlying molecular mechanisms of cancer development, we further analyzed co-mutation pattern of *ANK3* in KIRC. Genetic alterations of 124 genes were significantly identified in KIRC patients with *ANK3* mutations (Fig. 4C, Supplementary Table 1). Top 10 genes with the most significantly co-mutated with *ANK3* were *MCRS1* (microspherule protein 1), *SDAD1* (SDA1 domain containing 1), *TTN* (titin), *NFAT5* (nuclear factor of activated T cells 5), *PCLO* (piccolo presynaptic cytomatrix protein), *AJUBA* (Ajuba LIM protein), *NFASC* (neurofascin), *MSH6* (MutS homolog 6), *HOXA9* (homeobox A9), and *SLC7A6* (solute carrier family 7 member 6) (Fig. 4D). Among these genes, *SDAD1*, *NFAT5*, *PCLO*, *AJUBA*, and *MSH6* had a significant prognostic impact on overall survival for KIRC (Fig. 4E and 4F). These data suggested that mutations of *ANK3* and its co-mutated genes may involve cancer development and predict a high risk of poor prognosis in KIRC patients.

### Interaction network, prognostic impact, and functional enrichment of *ANK3*-correlated genes in KIRC

**Fig. 5.**
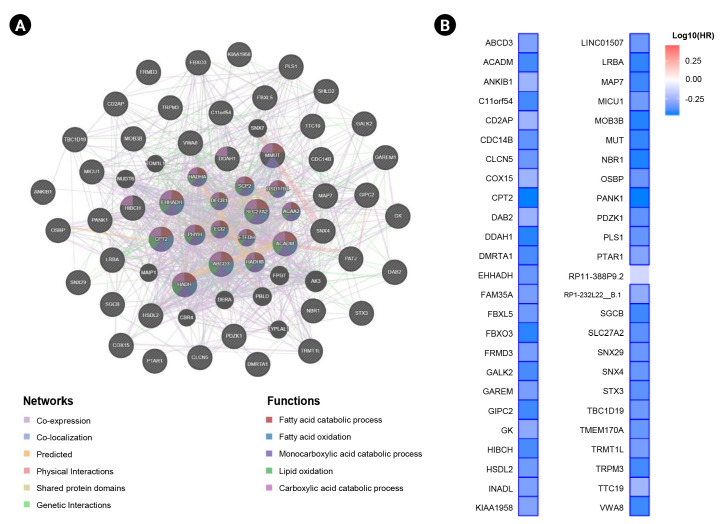
Interaction network and prognostic impact of *ANK3*-correlated genes in KIRC. (A) Interaction network of the top 50 genes with the highest correlation with *ANK3* in KIRC (GeneMANIA database). (B) Heatmap representing HR of each *ANK3*-correlated gene for overall survival of KIRC patients (GEPIA2 database). *ANK3*, ankyrin 3; KIRC, kidney renal clear cell carcinoma; HR, hazard ratio; GEPIA2, Gene Expression Profiling Interactive Analysis 2.

**Fig. 6.**
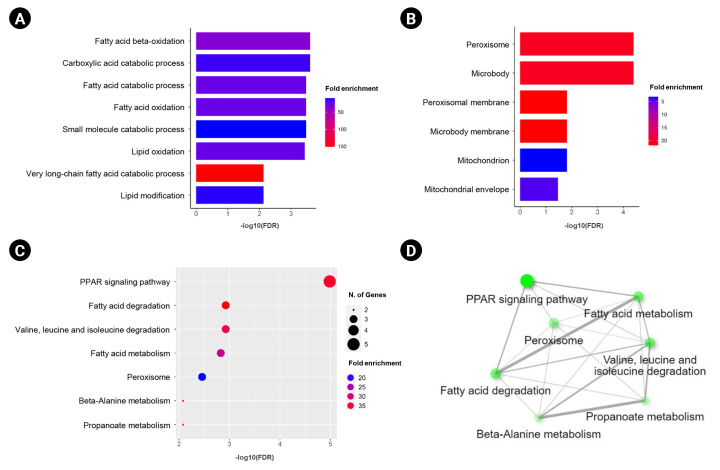
Functional enrichment of *ANK3*-correlated genes in KIRC. Bar graphs representing significantly enriched GO biological process terms (A) and GO cellular component terms (B) of *ANK3*-correlated genes in KIRC (ShinyGO database). (C, D) Dot plot and network of significantly enriched Kyoto Encyclopedia of Genes and Genomes pathway of *ANK3*-correlated genes in KIRC (ShinyGO database). *ANK3*, ankyrin 3; KIRC, kidney renal clear cell carcinoma; GO, gene ontology; FDR, false discovery rate.

In this study, we obtained the top 50 genes with the highest correlation with *ANK3* in KIRC dataset from GEPIA2 for further analyses to define the possible roles of *ANK3* in KIRC development and progression. A list of these genes is provided in Supplementary Table 2. The interaction network of *ANK3*-correlated genes was analyzed using GeneMANIA. As shown in Fig. 5A, these correlated genes closely interacted with each other in the network. The interactions among these genes were co-expression (91.98%), co-localization (3.53%), predicted (2.09%), physical interactions (1.85%), shared protein domains (0.3%), and genetic interactions (0.25%). Many of the genes in the network were significantly involved in several biological functions related to fatty acid and lipid metabolisms. Survival analysis using GEPIA2 revealed that most *ANK3*-correlated genes (49 of 50 genes) had a significant prognostic impact on overall survival for KIRC (Fig. 5B). In addition, functional enrichment analysis was also performed using ShinyGO. The data showed that these correlated genes were mainly enriched in GO biological process terms, such as “fatty acid beta-oxidation”, “carboxylic acid catabolic process”, and “fatty acid catabolic process” (Fig. 6A). The significantly enriched GO cellular component terms were predominantly involved with “peroxisome” and “microbody” (Fig. 6B). There was no significant enrichment of GO molecular function term in these correlated genes. For KEGG pathway enrichment analysis, *ANK3*-correlated genes were significantly enriched in several pathways, mainly including “PPAR signaling pathway”, “fatty acid degradation”, and “valine, leucine and isoleucine degradation” (Fig. 6C). These enriched pathways were closely connected with each other (Fig. 6D). Taken together, these findings suggested that *ANK3* and its correlated genes may play a role in KIRC through PPAR signaling pathways and lipid metabolism.

### Relationship between *ANK3* and PPAR genes in KIRC

**Fig. 7.**
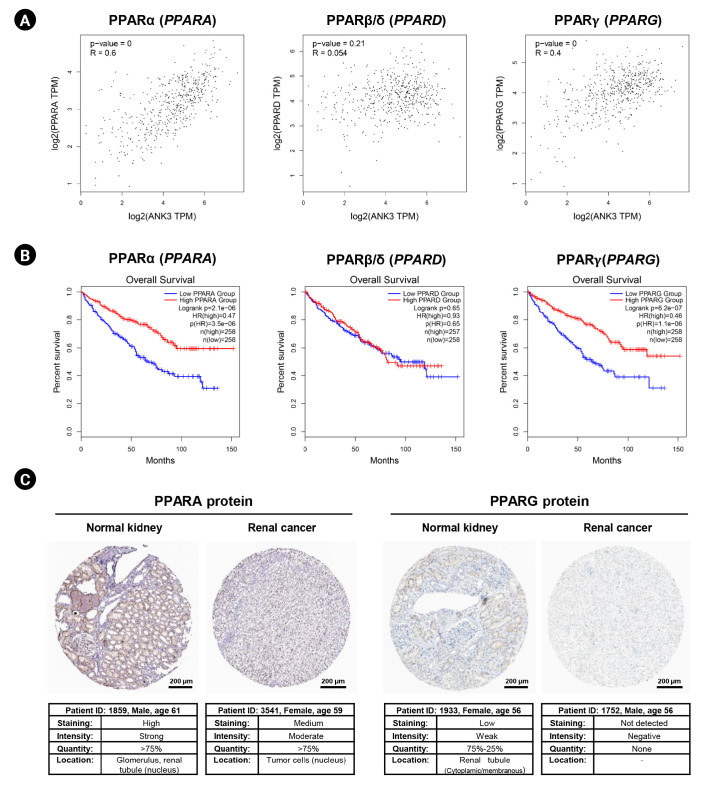
Correlation of PPAR expression with *ANK3* expression and patient’s overall survival in KIRC. (A) Scatter plots representing correlation of *ANK3* expression with *PPARA*, *PPARD*, and *PPARG* expressions in KIRC (GEPIA2 database). (B) KM curves for overall survival in KIRC patients with low- and high-expression of *PPARA*, *PPARD*, and *PPARG* (GEPIA2 database). (C) Representative immunohistochemical image of PPARA and PPARG protein expressions in normal kidney and renal cancer tissues (Human Protein Atlas database). PPAR, peroxisome proliferator-activated receptor; *ANK3*, ankyrin 3; KIRC, kidney renal clear cell carcinoma; GEPIA2, Gene Expression Profiling Interactive Analysis 2; KM, Kaplan-Meier; TPM, transcripts per million; HR, hazard ratio.

In order to explore a relationship between *ANK3* and PPAR signaling pathway in KIRC, we employed GEPIA2 to analyze the correlation between *ANK3* expression and three subfamilies of PPARs, including *PPARA*, *PPARD*, and *PPARG* in KIRC [30]. As shown in Fig. 7A, *ANK3* expression was significantly positively correlated with *PPARA* and *PPARG* expressions, but not correlated with *PPARD* expression in KIRC. Furthermore, survival analysis using GEPIA2 showed that KIRC patients with low expressions of *PPARA* and *PPARG* had significantly shorter overall survival compared to high-expression groups. There was no significant association between *PPARD* expression and overall survival in KIRC patients (Fig. 7B). The results from the HPA database confirmed a decrease of PPARA and PPARG protein in renal cancer compared to normal kidney tissues (Fig. 7C). These results demonstrated a possible relationship of *ANK3* to PPARα and PPARγ signaling pathways in KIRC pathogenesis and prognosis.

### Correlation between *ANK3* expression and immune cell infiltration in KIRC

**Fig. 8.**
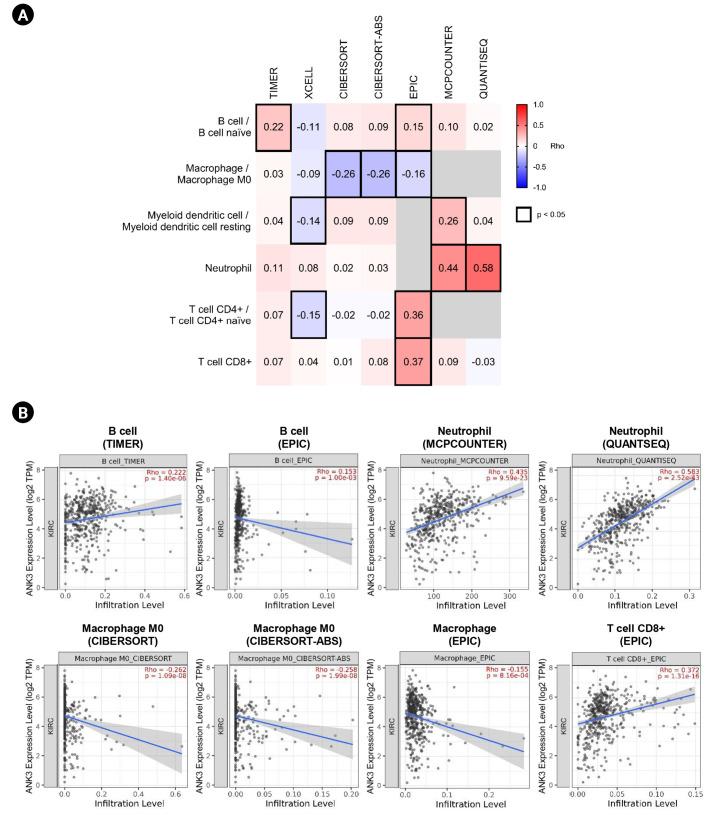
Correlation between *ANK3* expression and immune cell infiltration level in KIRC. (A) A heatmap representing the partial Spearman's correlation coefficient for correlation of *ANK3* expression with infiltration level of immune cells, estimated by different algorithms in the TIMER2.0 database with tumor purity adjustment. (B) Scatter plots representing significant correlation of *ANK3* expression and infiltration level of immune cells (TIMER2.0 database). *ANK3*, ankyrin 3; KIRC, kidney renal clear cell carcinoma; TIMER, Tumor Immune Estimation Resource; TPM, transcripts per million.

Because PPAR signaling pathway does not only involve energy homeostasis, but also plays a crucial role in regulating immune function and response in cancers [31,32]. Therefore, we further investigated the correlation between *ANK3* and immune cell infiltration in KIRC using TIMER2.0. As shown in Fig. 8, *ANK3* expression was consistently and significantly correlated with B cells, macrophages, neutrophils, and CD8+ T cells in KIRC. These findings suggested that *ANK3* expression was associated with abundance of tumor-infiltrating immune cells in KIRC tissue. The *ANK3* expression may be related to anti-tumor immunity and therapeutic responses in KIRC.

## Discussion

*ANK3* is the major form of ankyrin which is widely expressed in all nephron segments of the kidney [6,33]. It plays an important role in maintaining structural and physiological integrities of the kidney [7-9]. Recently, several studies have demonstrated that *ANK3* expression is positively associated with patient’s prognosis and exerts a tumor-suppressive function in many cancers [14-16]. Therefore, *ANK3* is an interesting target for further investigations on its prognostic value and role in KIRC.

In this study, our data analyses demonstrated that *ANK3* mRNA and protein expression levels were significantly decreased in KIRC compared to normal tissues. Decreased *ANK3* expression was positively correlated with disease stage and progression. The patients with low *ANK3* expression had poor survival outcomes. These findings indicated that *ANK3* expression had a favorable prognostic impact on KIRC. Our results are in line with previous studies in other types of cancer, including prostate and breast cancers [14,15], where low *ANK3* expression was associated with poor survival outcomes. Therefore, *ANK3* expression could serve as a predictive indicator for progression and prognosis in KIRC patients.

A number of genetic alterations have been described in KIRC. The von Hippel–Lindau (*VHL*) mutation is considered as an initiating factor for KIRC development [3]. Mutations in several genes, including polybromo 1 (*PBRM1*), SET domain containing 2 (*SETD2*), and BRCA-associated protein 1 (*BAP1*), are frequently identified and closely associated with the prognosis of KIRC [34]. To our knowledge, *ANK3* mutation and its functional impact on KIRC have not been previously reported. In this study, mutations were distributed throughout the *ANK3* gene. Among these, missense and frameshift mutations were found in ZU5 domain. It has been shown that the ZU5 domain of ANK3 serves as a binding site for β-spectrin to organize membrane components [35] and also plays a role in regulation of apoptosis [35,36]. These findings implied that *ANK3* mutations may affect its function in kidney homeostasis and carcinogenesis. *ANK3* mutations were found in only a small number of KIRC patients (2.4%), suggesting that such mutations might not directly influence *ANK3* expression level. Thus, epigenetic mechanisms could play a role in regulation of *ANK3* expression in KIRC and merit further studies. Because *ANK3* mutations occur at a very low frequency in KIRC patients, they may not have a direct association with prognosis. However, patients with *ANK3* mutations frequently carry additional mutations in several genes with strong favorable prognostic impact on overall survival for KIRC. Among these *ANK3*-comutated genes, mutations and loss of expression of *MSH6* have been reported and thought to be related to KIRC development [37,38]. In addition, *MSH6* has been identified as a predisposition gene in early-onset colorectal cancer and sporadic triple-negative breast cancer [39,40]. On the basis of these findings, it was suggested that mutations of *ANK3* and its co-mutated genes might involve cancer development and predict a high risk of a poor prognosis. However, further investigations are needed to confirm their clinical relevance in KIRC patients.

Previous studies have demonstrated that *ANK3* regulates various cellular processes in cancer cells, including cell cycle, apoptosis, and invasion [14-16]. Its tumor suppressive mechanisms are related to modulation of androgen receptor signaling pathway [14,15] and suppression of epithelial-mesenchymal transition process [16]. However, the precise role of *ANK3* and its mechanisms in KIRC carcinogenesis remains largely unknown. In this study, functional enrichment analyses of *ANK3*-correlated genes revealed potential involvement of *ANK3* in PPAR signaling pathways and lipid metabolism in KIRC. PPARs are nuclear receptor transcription factors which are classified into three main subfamilies: *PPARA*, *PPARD*, and *PPARG*. They play a major role in regulation of lipid metabolism and energy homeostasis [30]. Several lines of evidence have indicated that PPARs have a strong implication in cancers and have been recognized as promising therapeutic targets [31]. PPARα and PPARγ are widely considered to exert tumor suppressive function, whereas PPARβ/δ seems to play oncogenic role in many types of cancers [31]. In KIRC, PPARα and PPARγ have been shown to regulate tumor growth and metastasis via modulations of lipid and other metabolic pathways [41-44]; but there was no study reporting the role PPARβ/δ in this cancer. In concordance with these findings, our analyses revealed that *ANK3* expression was positively correlated with *PPARA* and *PPARG*, but not *PPARD* expression in KIRC. Low expressions of *PPARA* and *PPARG* were associated with poor prognosis in the patients. Decreased protein levels of PPARα and PPARγ were also confirmed in renal cancer tissue. Taken together, our results implied that PPARα and PPARγ play a more prominent role than PPARβ/δ in carcinogenesis and prognosis of KIRC. A previous study has reported that C-terminal region of ANK3 protein binds to sterol regulatory element-binding protein (SREBP), which is a transcriptional factor involved in regulation of fatty acid metabolism [45]. SREBP can activate PPARγ through stimulating the production of its endogenous ligand [46]. These findings suggested that *ANK3* might exert its tumor suppressive role in KIRC through modulation of PPARα and PPARγ pathways.

Emerging evidence indicates that PPARs play a crucial role in regulation of immune cell function and response [32]. Cancer progression, prognosis, and treatment outcomes of the patient with KIRC are strongly influenced by immune cells in tumor microenvironment [47-52]. Therefore, it was hypothesized that *ANK3* expression might be related to tumor-infiltrating immune cells in KIRC. In our study, we found a significant correlation between *ANK3* expression and infiltration level of various immune cells, including B cell, macrophage, neutrophils, and CD8+ T cell. However, there was a weak to moderate correlation observed from our analysis. Therefore, further experimental validations should be conducted to confirm the potential of *ANK3* as an indicator for immune infiltrate and response in KIRC.

To our knowledge, our study is the first to show the potential role of *ANK3* in prognosis and its possible relationships with PPARα/PPARγ signaling pathway and immune infiltration in KIRC. However, there are several limitations to this study that should be considered. First, we conducted bioinformatics analysis with a limited number of publicly available datasets. Although TCGA is a large and comprehensive dataset, it may not fully represent all KIRC patients. Future perspective and more independent cohort studies would help to confirm the prognostic significance of *ANK3* in KIRC. Second, further *in vitro*/*in vivo* experiments are needed to address the oncogenic role of *ANK3* and its mechanisms involved in regulating PPARα/PPARγ signaling pathways in KIRC. Finally, clinical relevance and underlying mechanism of *ANK3* in modulating immune response in KIRC requires further investigations.

In conclusion, our findings demonstrated the prognostic significance of *ANK3* and its potential involvement with PPARα/PPARγ signaling pathway and immune cell infiltration in KIRC. *ANK3* could serve as a prognostic biomarker and promising therapeutic target for KIRC.

## Figures and Tables

**Table 1.** Multivariable Cox proportional hazard regression analysis of factors affecting overall survival of KIRC patients (TIMER database)

Variable	HR (95% CI)	p-value
Age	1.029 (1.015–1.044)	<0.001
Sex (male)	0.837 (0.607–1.154)	0.278
Race (Black)	2.235 (0.282–17.736)	0.447
Race (White)	2.142 (0.293–15.664)	0.453
Stage2	1.203 (0.644 - 2.247)	0.562
Stage3	2.172 (1.427–3.305)	<0.001
Stage4	6.257 (4.237–9.238)	<0.001
*ANK3* expression	0.739 (0.669–0.816)	<0.001

KIRC, kidney renal clear cell carcinoma; TIMER, Tumor Immune Estimation Resource; HR, hazard ratio; CI, confidence interval; *ANK3*, ankyrin 3.

## Supplementary Materials

Supplementary Table 1. List of mutated genes that were significantly enriched in kidney renal clear cell carcinoma patients with *ANK3* mutations (cBioPortal database)

Supplementary Table 2. List of the top 50 genes that were positively correlated with *ANK3* in kidney renal clear cell carcinoma (GEPIA2 database)
