# Supplementary material for: Bioinformatic analyses reveal the prognostic significance and potential role of ankyrin 3 (ANK3) in kidney renal clear cell carcinoma
Source: Genomics Inform. 2023 Jun 30;21(2):e22. doi: 10.5808/gi.23013 (PMC10326534; doi:10.5808/gi.23013)
Supplement: Supplementary Table 1. — List of mutated genes that were significantly enriched in kidney renal clear cell carcinoma patients with ANK3 mutations (cBioPortal database) [file gi-23013-Supplementary-Table-1.pdf]

Supplementary Materials – Table 1

Bioinformatic analyses reveal the prognostic significance and potential role of ankyrin 3 (*ANK3*) in kidney renal clear cell carcinoma

Keerakarn Somsuan and Siripat Aluksanasuwan

Correspondence to Siripat Aluksanasuwan; siripat.alu@mfu.ac.th

Supplementary Table 1. List of mutated genes that were significantly enriched in kidney renal clear cell carcinoma patients with *ANK3* mutations (cBioPortal database)

No.	Gene	Cytoband	Altered group	Unaltered group	Log ratio	p-value	q-value	Enriched in
1	*MCRS1*	12q13.12	5 (13.89%)	0 (0.00%)	>10	6.26E-09	1.30E-04	Altered group
2	*SDAD1*	4q21.1	6 (16.67%)	10 (0.69%)	4.6	8.81E-07	8.66E-03	Altered group
3	*TTN*	2q31.2	17 (47.22%)	195 (13.44%)	1.81	1.59E-06	8.66E-03	Altered group
4	*NFAT5*	16q22.1	6 (16.67%)	12 (0.83%)	4.33	1.97E-06	8.66E-03	Altered group
5	*PCLO*	7q21.11	9 (25.00%)	44 (3.03%)	3.04	2.09E-06	8.66E-03	Altered group
6	*AJUBA*	14q11.2	5 (13.89%)	6 (0.41%)	5.07	2.60E-06	8.99E-03	Altered group
7	*NFASC*	1q32.1	7 (19.44%)	23 (1.59%)	3.62	3.63E-06	9.59E-03	Altered group
8	*MSH6*	2p16.3	5 (13.89%)	7 (0.48%)	4.85	4.39E-06	9.59E-03	Altered group
9	*HOXA9*	7p15.2	3 (11.54%)	0 (0.00%)	>10	4.85E-06	9.59E-03	Altered group
10	*SLC7A6*	16q22.1	3 (11.54%)	0 (0.00%)	>10	4.85E-06	9.59E-03	Altered group
11	*TDRD1*	10q25.3	5 (13.89%)	1 (0.19%)	6.17	5.09E-06	9.59E-03	Altered group
12	*PPP4R3A*	14q32.12	5 (13.89%)	9 (0.62%)	4.48	1.07E-05	1.08E-02	Altered group
13	*AMER3*	2q21.1	3 (8.33%)	0 (0.00%)	>10	1.31E-05	1.08E-02	Altered group
14	*BAAT*	9q31.1	3 (8.33%)	0 (0.00%)	>10	1.31E-05	1.08E-02	Altered group
15	*CIZ1*	9q34.11	3 (8.33%)	0 (0.00%)	>10	1.31E-05	1.08E-02	Altered group
16	*DUSP29*	10q22.2	3 (8.33%)	0 (0.00%)	>10	1.31E-05	1.08E-02	Altered group
17	*GFOD1*	6p24.1-p23	3 (8.33%)	0 (0.00%)	>10	1.31E-05	1.08E-02	Altered group
18	*HCAR2*	12q24.31	3 (8.33%)	0 (0.00%)	>10	1.31E-05	1.08E-02	Altered group
19	*HLA-DRA*	6p21.32	3 (8.33%)	0 (0.00%)	>10	1.31E-05	1.08E-02	Altered group
20	*LYSMD4*	15q26.3	3 (8.33%)	0 (0.00%)	>10	1.31E-05	1.08E-02	Altered group
21	*MAPK13*	6p21.31	3 (8.33%)	0 (0.00%)	>10	1.31E-05	1.08E-02	Altered group
22	*METTL25*	12q21.31	3 (8.33%)	0 (0.00%)	>10	1.31E-05	1.08E-02	Altered group
23	*MRPL30*	2q11.2	3 (8.33%)	0 (0.00%)	>10	1.31E-05	1.08E-02	Altered group
24	*PLPP3*	1p32.2	3 (8.33%)	0 (0.00%)	>10	1.31E-05	1.08E-02	Altered group
25	*PRPH*	12q13.12	3 (8.33%)	0 (0.00%)	>10	1.31E-05	1.08E-02	Altered group
26	*ANKRD11*	16q24.3	5 (13.89%)	10 (0.69%)	4.33	1.58E-05	1.17E-02	Altered group
27	*NLRC5*	16q13	5 (13.89%)	10 (0.69%)	4.33	1.58E-05	1.17E-02	Altered group
28	*ZFYVE27*	10q24.2	5 (13.89%)	10 (0.69%)	4.33	1.58E-05	1.17E-02	Altered group
29	*SGO2*	2q33.1	6 (16.67%)	19 (1.31%)	3.67	1.66E-05	1.19E-02	Altered group
30	*SLC9B2*	4q24	4 (11.11%)	4 (0.28%)	5.33	1.90E-05	1.31E-02	Altered group
31	*WWP2*	16q22.1	4 (15.38%)	4 (0.42%)	5.19	2.57E-05	1.72E-02	Altered group
32	*TMEM81*	1q32.1	4 (11.11%)	5 (0.34%)	5.01	3.35E-05	2.17E-02	Altered group
33	*FAT3*	11q14.3	7 (19.44%)	35 (2.41%)	3.01	3.90E-05	2.45E-02	Altered group
34	*BLTP3A*	6p21.31	5 (13.89%)	13 (0.90%)	3.95	4.27E-05	2.53E-02	Altered group
35	*TEC*	4p12-p11	5 (13.89%)	13 (0.90%)	3.95	4.27E-05	2.53E-02	Altered group

36	*NPR2*	9p13.3	4 (11.11%)	3 (0.32%)	5.14	4.83E-05	2.78E-02	Altered group
37	*HECW2*	2q32.3	6 (16.67%)	24 (1.65%)	3.33	5.11E-05	2.85E-02	Altered group
38	*NCAPH2*	22q13.33	4 (11.11%)	6 (0.41%)	4.75	5.49E-05	2.85E-02	Altered group
39	*UNC13B*	9p13.3	4 (11.11%)	6 (0.41%)	4.75	5.49E-05	2.85E-02	Altered group
40	*PDE4C*	19p13.11	5 (13.89%)	14 (0.96%)	3.85	5.69E-05	2.85E-02	Altered group
41	*REV3L*	6q21	5 (19.23%)	21 (1.45%)	3.73	5.78E-05	2.85E-02	Altered group
42	*TNC*	9q33.1	5 (19.23%)	21 (1.45%)	3.73	5.78E-05	2.85E-02	Altered group
43	*CELSR2*	1p13.3	5 (13.89%)	15 (1.03%)	3.75	7.46E-05	3.43E-02	Altered group
44	*PLEKHA6*	1q32.1	5 (13.89%)	15 (1.03%)	3.75	7.46E-05	3.43E-02	Altered group
45	*SPAM1*	7q31	5 (13.89%)	15 (1.03%)	3.75	7.46E-05	3.43E-02	Altered group
46	*ATP8B1*	18q21.31	4 (11.11%)	7 (0.48%)	4.53	8.48E-05	3.66E-02	Altered group
47	*BIN2*	12q13.13	4 (11.11%)	7 (0.48%)	4.53	8.48E-05	3.66E-02	Altered group
48	*POLG*	15q26.1	4 (11.11%)	7 (0.48%)	4.53	8.48E-05	3.66E-02	Altered group
49	*MYH8*	17p13.1	4 (15.38%)	11 (0.76%)	4.34	9.05E-05	3.83E-02	Altered group
50	*ZBED4*	22q13.33	3 (11.54%)	3 (0.21%)	5.8	9.37E-05	3.88E-02	Altered group
51	*DNAJB11*	3q27.3	5 (13.89%)	16 (1.10%)	3.65	9.62E-05	3.91E-02	Altered group
52	*KIRREL1*	1q23.1	5 (13.89%)	17 (1.17%)	3.57	1.22E-04	4.53E-02	Altered group
53	*POLE*	12q24.33	5 (13.89%)	17 (1.17%)	3.57	1.22E-04	4.53E-02	Altered group
54	*ZNF687*	1q21.3	5 (13.89%)	17 (1.17%)	3.57	1.22E-04	4.53E-02	Altered group
55	*CASQ1*	1q23.2	4 (11.11%)	8 (0.55%)	4.33	1.25E-04	4.53E-02	Altered group
56	*TRIM51*	11q12.1	4 (11.11%)	8 (0.55%)	4.33	1.25E-04	4.53E-02	Altered group
57	*DLG5*	10q22.3	3 (12.50%)	2 (0.21%)	5.89	1.27E-04	4.53E-02	Altered group
58	*PTGFR*	1p31.1	3 (12.50%)	2 (0.21%)	5.89	1.27E-04	4.53E-02	Altered group
59	*DIO2*	14q31.1	4 (11.11%)	9 (0.62%)	4.16	1.78E-04	4.98E-02	Altered group
60	*ERBIN*	5q12.3	4 (11.11%)	9 (0.62%)	4.16	1.78E-04	4.98E-02	Altered group
61	*FLT3*	13q12.2	4 (11.11%)	9 (0.62%)	4.16	1.78E-04	4.98E-02	Altered group
62	*ZC3H13*	13q14.13	4 (11.11%)	9 (0.62%)	4.16	1.78E-04	4.98E-02	Altered group
63	*AHNAK2*	14q32.33	7 (19.44%)	46 (3.17%)	2.62	1.84E-04	4.98E-02	Altered group
64	*CES1P1*	16q12.2	2 (9.09%)	0 (0.00%)	>10	2.13E-04	4.98E-02	Altered group
65	*SLC8B1*	12q24.13	5 (13.89%)	13 (1.28%)	3.44	2.20E-04	4.98E-02	Altered group
66	*CFAP61*	20p11.23	4 (11.11%)	10 (0.69%)	4.01	2.44E-04	4.98E-02	Altered group
67	*HPS5*	11p15.1	4 (11.11%)	10 (0.69%)	4.01	2.44E-04	4.98E-02	Altered group
68	*SLC7A13*	8q21.3	4 (11.11%)	10 (0.69%)	4.01	2.44E-04	4.98E-02	Altered group
69	*COL14A1*	8q24.12	4 (15.38%)	15 (1.03%)	3.9	2.45E-04	4.98E-02	Altered group
70	*ADORA2A*	22q11.23	3 (8.33%)	3 (0.21%)	5.33	2.48E-04	4.98E-02	Altered group
71	*ATXN2*	12q24.12	3 (8.33%)	3 (0.21%)	5.33	2.48E-04	4.98E-02	Altered group
72	*CACNB1*	17q12	3 (8.33%)	3 (0.21%)	5.33	2.48E-04	4.98E-02	Altered group
73	*DNM1L*	12p11.21	3 (8.33%)	3 (0.21%)	5.33	2.48E-04	4.98E-02	Altered group
74	*DTNBP1*	6p22.3	3 (8.33%)	3 (0.21%)	5.33	2.48E-04	4.98E-02	Altered group
75	*FAM166B*	9p13.3	3 (8.33%)	3 (0.21%)	5.33	2.48E-04	4.98E-02	Altered group
76	*FERMT3*	11q13.1	3 (8.33%)	3 (0.21%)	5.33	2.48E-04	4.98E-02	Altered group
77	*FMNL3*	12q13.12	3 (8.33%)	3 (0.21%)	5.33	2.48E-04	4.98E-02	Altered group
78	*FNBP1L*	1p22.1	3 (8.33%)	3 (0.21%)	5.33	2.48E-04	4.98E-02	Altered group
79	*GRK3*	22q12.1	3 (8.33%)	3 (0.21%)	5.33	2.48E-04	4.98E-02	Altered group
80	*GTSE1*	22q13.31	3 (8.33%)	3 (0.21%)	5.33	2.48E-04	4.98E-02	Altered group
81	*HUS1*	7p12.3	3 (8.33%)	3 (0.21%)	5.33	2.48E-04	4.98E-02	Altered group
82	*INTS11*	1p36.33	3 (8.33%)	3 (0.21%)	5.33	2.48E-04	4.98E-02	Altered group
83	*RSC1A1*	1p36.21	3 (8.33%)	3 (0.21%)	5.33	2.48E-04	4.98E-02	Altered group

84	*TCERG1L*	10q26.3	3 (8.33%)	3 (0.21%)	5.33	2.48E-04	4.98E-02	Altered group
85	*TNFRSF9*	1p36.23	3 (8.33%)	3 (0.21%)	5.33	2.48E-04	4.98E-02	Altered group
86	*TSPAN11*	12p11.21	3 (8.33%)	3 (0.21%)	5.33	2.48E-04	4.98E-02	Altered group
87	*TULP1*	6p21.31	3 (8.33%)	3 (0.21%)	5.33	2.48E-04	4.98E-02	Altered group
88	*UBXN10*	1p36.12	3 (8.33%)	3 (0.21%)	5.33	2.48E-04	4.98E-02	Altered group
89	*ZGLP1*	19p13.2	3 (8.33%)	3 (0.21%)	5.33	2.48E-04	4.98E-02	Altered group
90	*ZNF215*	11p15.4	3 (8.33%)	3 (0.21%)	5.33	2.48E-04	4.98E-02	Altered group
91	*ZNF358*	19p13.2	3 (8.33%)	3 (0.21%)	5.33	2.48E-04	4.98E-02	Altered group
92	*LZTR1*	22q11.21	2 (8.33%)	0 (0.00%)	>10	2.54E-04	4.98E-02	Altered group
93	*RSAD2*	2p25.2	2 (8.33%)	0 (0.00%)	>10	2.54E-04	4.98E-02	Altered group
94	*FAT4*	4q28.1	5 (13.89%)	21 (1.45%)	3.26	2.85E-04	4.98E-02	Altered group
95	*SLITRK6*	13q31.1	5 (13.89%)	21 (1.45%)	3.26	2.85E-04	4.98E-02	Altered group
96	*ADA2*	22q11.1	3 (21.43%)	12 (0.83%)	4.7	2.96E-04	4.98E-02	Altered group
97	*ACVR1*	2q24.1	2 (7.69%)	0 (0.00%)	>10	2.98E-04	4.98E-02	Altered group
98	*BAG1*	9p13.3	2 (7.69%)	0 (0.00%)	>10	2.98E-04	4.98E-02	Altered group
99	*CFAP299*	4q21.21	2 (7.69%)	0 (0.00%)	>10	2.98E-04	4.98E-02	Altered group
100	*CLEC18A*	16q22.1	2 (7.69%)	0 (0.00%)	>10	2.98E-04	4.98E-02	Altered group
101	*CLEC18C*	16q22.1	2 (7.69%)	0 (0.00%)	>10	2.98E-04	4.98E-02	Altered group
102	*DDX19B*	16q22.1	2 (7.69%)	0 (0.00%)	>10	2.98E-04	4.98E-02	Altered group
103	*DIS3L*	15q22.31	2 (7.69%)	0 (0.00%)	>10	2.98E-04	4.98E-02	Altered group
104	*DMAP1*	1p34.1	2 (7.69%)	0 (0.00%)	>10	2.98E-04	4.98E-02	Altered group
105	*DPCD*	10q24.32	2 (7.69%)	0 (0.00%)	>10	2.98E-04	4.98E-02	Altered group
106	*DTX3*	12q13.3	2 (7.69%)	0 (0.00%)	>10	2.98E-04	4.98E-02	Altered group
107	*ENOSF1*	18p11.32	2 (7.69%)	0 (0.00%)	>10	2.98E-04	4.98E-02	Altered group
108	*EXOSC6*	16q22.1	2 (7.69%)	0 (0.00%)	>10	2.98E-04	4.98E-02	Altered group
109	*GLO1*	6p21.2	2 (7.69%)	0 (0.00%)	>10	2.98E-04	4.98E-02	Altered group
110	*GRK4*	4p16.3	2 (7.69%)	0 (0.00%)	>10	2.98E-04	4.98E-02	Altered group
111	*HOXC11*	12q13.13	2 (7.69%)	0 (0.00%)	>10	2.98E-04	4.98E-02	Altered group
112	*MAPK7*	17p11.2	2 (7.69%)	0 (0.00%)	>10	2.98E-04	4.98E-02	Altered group
113	*MBOAT1*	6p22.3	2 (7.69%)	0 (0.00%)	>10	2.98E-04	4.98E-02	Altered group
114	*METAP2*	12q22	2 (7.69%)	0 (0.00%)	>10	2.98E-04	4.98E-02	Altered group
115	*MPV17*	2p23.3	2 (7.69%)	0 (0.00%)	>10	2.98E-04	4.98E-02	Altered group
116	*NQO1*	16q22.1	2 (7.69%)	0 (0.00%)	>10	2.98E-04	4.98E-02	Altered group
117	*OR4L1*	14q11.2	2 (7.69%)	0 (0.00%)	>10	2.98E-04	4.98E-02	Altered group
118	*PDLIM1*	10q23.33	2 (7.69%)	0 (0.00%)	>10	2.98E-04	4.98E-02	Altered group
119	*SNAPC5*	15q22.31	2 (7.69%)	0 (0.00%)	>10	2.98E-04	4.98E-02	Altered group
120	*SPNS2*	17p13.2	2 (7.69%)	0 (0.00%)	>10	2.98E-04	4.98E-02	Altered group
121	*THTPA*	14q11.2	2 (7.69%)	0 (0.00%)	>10	2.98E-04	4.98E-02	Altered group
122	*TNFAIP8L3*	15q21.2	2 (7.69%)	0 (0.00%)	>10	2.98E-04	4.98E-02	Altered group
123	*UROD*	1p34.1	2 (7.69%)	0 (0.00%)	>10	2.98E-04	4.98E-02	Altered group
124	*VIM*	10p13	2 (7.69%)	0 (0.00%)	>10	2.98E-04	4.98E-02	Altered group
