# Supplementary material for: Bioinformatic analyses reveal the prognostic significance and potential role of ankyrin 3 (ANK3) in kidney renal clear cell carcinoma
Source: Genomics Inform. 2023 Jun 30;21(2):e22. doi: 10.5808/gi.23013 (PMC10326534; doi:10.5808/gi.23013)
Supplement: Supplementary Table 2. — List of the top 50 genes that were positively correlated with ANK3 in kidney renal clear cell carcinoma (GEPIA2 database) [file gi-23013-Supplementary-Table-2.pdf]

Supplementary Materials – Table 2

Bioinformatic analyses reveal the prognostic significance and potential role of ankyrin 3 (*ANK3*) in kidney renal clear cell carcinoma

Keerakarn Somsuan and Siripat Aluksanasuwan

Correspondence to Siripat Aluksanasuwan; siripat.alu@mfu.ac.th

Supplementary Table 2. List of the top 50 genes that were positively correlated with *ANK3* in kidney renal clear cell carcinoma (GEPIA2 database)

No.	Gene symbol	Gene ID	Description	Pearson correlation coefficient
1	*CLCN5*	ENSG00000171365.15	Chloride voltage-gated channel 5	0.72
2	*HSDL2*	ENSG00000119471.14	Hydroxysteroid dehydrogenase like 2	0.71
3	*TBC1D19*	ENSG00000109680.10	TBC1 domain family member 19	0.70
4	*ANKIB1*	ENSG00000001629.9	Ankyrin repeat and IBR domain containing 1	0.70
5	*MICU1*	ENSG00000107745.16	Mitochondrial calcium uptake 1	0.69
6	*MUT*	ENSG00000146085.7	Methylmalonyl-CoA mutase	0.69
7	*MAP7*	ENSG00000135525.18	Microtubule associated protein 7	0.68
8	*FBXO3*	ENSG00000110429.13	F-box protein 3	0.68
9	*STX3*	ENSG00000166900.14	Syntaxin 3	0.67
10	*PANK1*	ENSG00000152782.16	Pantothenate kinase 1	0.67
11	*ACADM*	ENSG00000117054.13	Acyl-CoA dehydrogenase medium chain	0.66
12	*GAREM*	ENSG00000141441.15	GRB2 associated regulator of MAPK1 subtype 1	0.66
13	*RP11-388P9.2*	ENSG00000232682.2	Novel transcript, antisense to ANK3	0.66
14	*COX15*	ENSG00000014919.12	Cytochrome c oxidase assembly homolog COX15	0.66
15	*SNX4*	ENSG00000114520.10	Sorting nexin 4	0.66
16	*MOB3B*	ENSG00000120162.9	MOB kinase activator 3B	0.66
17	*GK*	ENSG00000198814.12	Glycerol kinase	0.65
18	*EHHADH*	ENSG00000113790.10	Enoyl-CoA hydratase and 3-hydroxyacyl CoA dehydrogenase	0.65
19	*FBXL5*	ENSG00000118564.14	F-box and leucine rich repeat protein 5	0.65
20	*SGCB*	ENSG00000163069.12	Sarcoglycan beta	0.65
21	*RP1-232L22__B.1*	ENSG00000178146.9	Glycerol kinase 4 pseudogene	0.64
22	*CD2AP*	ENSG00000198087.7	CD2 associated protein	0.64
23	*C11orf54*	ENSG00000182919.14	Chromosome 11 open reading frame 54	0.64
24	*HIBCH*	ENSG00000198130.14	3-hydroxyisobutyryl-CoA hydrolase	0.64
25	*GALK2*	ENSG00000156958.14	Galactokinase 2	0.64
26	*PDZK1*	ENSG00000174827.13	PATJ crumbs cell polarity complex component	0.64
27	*LRBA*	ENSG00000198589.10	LPS responsive beige-like anchor protein	0.63
28	*PLS1*	ENSG00000120756.12	Plastin 1	0.63
29	*SLC27A2*	ENSG00000140284.10	Solute carrier family 27 member 2	0.63
30	*FRMD3*	ENSG00000172159.15	FERM domain containing 3	0.63

31	*GIPC2*	ENSG00000137960.5	GIPC PDZ domain containing family member 2	0.63
32	*FAM35A*	ENSG00000122376.11	Shieldin complex subunit 2	0.63
33	*DMRTA1*	ENSG00000176399.3	DMRT like family A1	0.63
34	*TMEM170A*	ENSG00000166822.12	Transmembrane protein 170A	0.63
35	*CPT2*	ENSG00000157184.5	Carnitine palmitoyltransferase 2	0.63
36	*TTC19*	ENSG00000011295.15	Tetratricopeptide repeat domain 19	0.63
37	*ABCD3*	ENSG00000117528.11	ATP binding cassette subfamily D member 3	0.62
38	*CDC14B*	ENSG00000081377.16	Cell division cycle 14B	0.62
39	*TRPM3*	ENSG00000083067.22	Transient receptor potential cation channel subfamily M member 3	0.62
40	*VWA8*	ENSG00000102763.15	Von Willebrand factor A domain containing 8	0.62
41	*DDAH1*	ENSG00000153904.18	Dimethylarginine dimethylaminohydrolase 1	0.62
42	*PTAR1*	ENSG00000188647.12	Protein prenyltransferase alpha subunit repeat containing 1	0.62
43	*NBR1*	ENSG00000188554.13	NBR1 autophagy cargo receptor	0.62
44	*OSBP*	ENSG00000110048.11	Oxysterol binding protein	0.62
45	*LINC01507*	ENSG00000230945.1	Long intergenic non-protein coding RNA 1507	0.62
46	*KIAA1958*	ENSG00000165185.14	KIAA1958	0.62
47	*INADL*	ENSG00000132849.18	PATJ crumbs cell polarity complex component	0.62
48	*TRMT1L*	ENSG00000121486.11	tRNA methyltransferase 1 like	0.62
49	*SNX29*	ENSG00000048471.13	Sorting nexin 29	0.62
50	*DAB2*	ENSG00000153071.14	DAB adaptor protein 2	0.62
